# Biomechanical Analysis of Post–Core Crown Restoration in Dog Maxillary Canine Teeth

**DOI:** 10.3390/vetsci13060514

**Published:** 2026-05-26

**Authors:** Mingfei Ding, Huanhuan Li, Min Yang, Ruixue Chen, Siyu Zhang, Jie Yang, Haifeng Liu, Zhijun Zhong, Guangneng Peng, Chengli Zheng, Ming Zhang, Ziyao Zhou

**Affiliations:** 1 College of Veterinary Medicine, Sichuan Agricultural University, Chengdu 611130, China; 2 Wangmiaotai Animal Hospital, Chengdu 610065, China; 3 Pet Nutrition and Health Research Center, Chengdu Agricultural College, Chengdu 611130, China; 4 Sichuan Institute of Musk Deer Breeding, Sichuan Institute for Drug Control, Chengdu 611731, China; 5 College of Animal Science and Technology, Sichuan Agricultural University, Chengdu 611130, China

**Keywords:** tooth fracture, canine teeth, post–core restoration, finite element analysis, computer simulation

## Abstract

Post–core restoration serves as an effective treatment for fractured canine teeth. However, the influence of post length on the stability of the teeth for small animals remains controversial. This study employs finite element analysis and a force machine in vitro to evaluate the biomechanical effects of varying fiber post lengths in maxillary canine teeth, aiming to establish theoretical guidelines for optimizing post–core restoration in canine dentistry. Three-dimensional models of the canine teeth of large, medium, and small dogs (German Shepherds, Beagles, and Teddy dogs) were constructed using finite element analysis (FEA). Fiber posts with post-to-root length ratios of 1/3, 1/2, and 2/3 were established. Stress distribution (σ_1_/σ_2_) and total deformation under 100–1100 N loading were analyzed virtually in ANSYS (version 17.0.0.19190), as well as in a force machine in vitro. FEA and in vitro fracture tests showed good correlation (*p* > 0.05). Fracture loads for large, medium, and small breeds were 1115.851 ± 6.984 N (distal) and 1177.39 ± 5.82 N (lingual), 901.627 ± 7.49 N (distal) and 976.504 ± 6.399 N (lingual), and 812.733 ± 5.476 N (distal) and 897.642 ± 6.42 N (lingual), respectively. The fiber post with a root–post ratio of 2/3 exhibited the highest fracture resistance, potentially making it be the best choice for post–core restoration.

## Simple Summary

Dog canine teeth are very important for biting, chewing and self-defense. When a tooth is broken at the root, a common treatment is to use a fiber post and crown to repair it. However, there are very few studies about the best length of the fiber post for dog rather than human teeth. In this study, we used computer modeling and lab tests to compare different sizes of dogs, as well as different lengths of fiber posts, to determine the best procedure. The results showed that using a fiber post with a 2/3 tooth root length might make the repaired tooth the strongest. Our study is the first to investigate the mechanics of root post repair in the veterinary field, whose findings give clear veterinary guidelines for fixing broken canine teeth in dogs.

## 1. Introduction

Teeth are crucial for the health and quality of life of animals. In dogs, the canine teeth primarily function in offense, hunting, and food tearing, making them some of the most critical functional teeth in the oral cavity. However, the canine teeth have a high fracture rate of up to 4.73% to 59.9% [1,2]. When a tooth is broken in the root, current restorative methods, such as root canal therapy or dental restoration, may not have enough biomechanical force between the restoration and the natural tooth, in cases of tooth root fractures.

Post–core restoration is mainly used to treat teeth with extensive coronal defects, and involves placing a post into the root canal space to provide support and retention for the core buildup, followed by the addition of a core foundation to serve as the base for the final restoration (typically a crown) [3]. The use of a post–core approach, however, requires the removal of additional tooth structure and thus weakens the remaining root dentin. Nevertheless, it remains an indispensable clinical procedure to provide a stable and durable framework for subsequent crown restoration, which is essential for restoring the masticatory function of endodontically treated teeth [4]. Due to the superior mechanical properties for clinical applications—including tensile strength, stiffness, resistance to biochemical degradation, low solubility, and minimal toxicity—fiber posts might be the preferred clinical choice for small animals. However, the impact of fiber post length on post-restoration stability in small animals remains controversial. Some studies suggest that increasing post length significantly improves retention and fracture resistance [5], advocating for posts to be as long as possible without compromising apical seal [6,7]. Conversely, other research has found no statistical correlation between post length and restoration stability [8].

Recent advances in finite element analysis in human dentistry have enabled accurate prediction of stress distribution in restored teeth [9], but veterinary applications remain limited. Furthermore, the influence of post length on fracture resistance in canine teeth with different crown–root morphologies in real teeth is poorly understood. Therefore, the objective of this study was to: (1) compare the biomechanical performance of three post-to-root length ratios (1/3, 1/2, 2/3) in maxillary canine teeth of large, medium, and small dogs using FEA; (2) validate the FEA predictions with in vitro fracture tests; and (3) provide evidence-based recommendations for clinical post selection in veterinary dentistry.

In this study, we first established 3D models of upper canines from three dog breeds (German Shepherds, Beagles, and Teddy dogs) using Computed Tomography (CT) scans. The intact and post–core crown-restored canine models were constructed in Mimics, Geomagic, and SOLIDWORKS, then imported into ANSYS for mechanical analysis. Maximum principal stress, minimum principal stress, and total deformation cloud maps were analyzed to provide a theoretical basis for post–core restoration in dogs. Our study is the first to investigate the mechanics of root post repair in the veterinary field, whose findings give veterinary clear guidelines for fixing broken canine teeth in dogs.

## 2. Materials and Methods

### 2.1. Ethics Statement

This study was approval by the Sichuan Agricultural University Animal Ethical and Welfare Committee (20240221, date of approval 5 March 2024).

### 2.2. Data Acquisition of Canine Teeth in German Shepherds, Beagles, and Teddy Dogs

**Figure 1:**
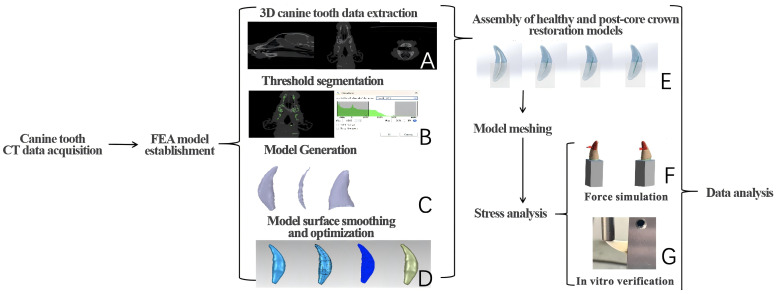
Scheme of computer simulation of tooth fracture. 3Dcaninetoothdata extraction (**A**) Threshold segmentation (**B**) ModelGeneration (**C**) Model surface smoothing and optimization (**D**) Assembly of healthy and post-code crown restoration models (**E**) Force simulation (**F**) Im vitro verification (**G**).

Healthy adult large (German Shepherd), medium (Beagle), and small (Teddy) dogs with fully formed canines and without wear or oral diseases were selected. CT scans (uCT503e, 40-slice detector, 40 slices, Chengdu Ultrasound Imaging Center Co., Ltd., Chengdu, China) were performed using the following parameters: voltage: 120 kV, current: 250 mA, reconstruction matrix: 512 × 512, slice thickness: 1 mm, bone window reconstruction algorithm with a window width of 2600 HU and a window position of 800 HU, and a soft tissue window reconstruction algorithm with a window width of 300 HU and a window position of 40 HU [10]. The acquired DICOM (Digital Imaging and Communications in Medicine) files were imported into Mimics Medical (Materialise Co., Ltd., Leuven, Belgium, Version 21.0.406) (Figure 1A).

### 2.3. Establishment of Healthy Canine Tooth Models

In Mimics, a new mask was first created through the “New Mask” function with appropriate threshold adjustment to select target images (Figure 1B). As the initially extracted model contained the entire skull structure, the “Edit Mask” tool was employed to refine the region of dental tissues (dentin, pulp, and enamel) and surrounding bone. Following a comprehensive 3D visual inspection, initial surface smoothing was applied via the “Smooth” function [11]. The finalized models were systematically exported in STL format, yielding a total of nine discrete models for subsequent analysis (Figure 1C).

### 2.4. Optimizing of the Quality and Appearance of Models

The STL files were imported into Geomagic Wrap (version 2021.0.0.3008), where initial mesh refinement was performed in the “Polygon” module using the “Remesh” function to optimize the nine models. This process improved mesh quality by achieving more uniform, standardized, and anatomically accurate grid structures. Subsequent application of the “Relax” and “Denoise” functions enhanced surface curvature and sharpness while eliminating data noise and irregularities, resulting in smoother, continuous models that retained original anatomical features. Problematic mesh areas were manually corrected using the “Remove Features” function to optimize both quality (through density adjustment and unnecessary detail removal) and appearance (via hole inspection/repair and surface smoothing). Model integrity was verified using the “Mesh Doctor” function until no error alerts were generated. The processed models underwent advanced surface reconstruction by the “Construct Patches” function and “Build Lattice” function. Finally, the “Fit Surfaces” function integration perfectly matched lattice and model surfaces, converting them to solids [12]. This comprehensive workflow enhanced geometric accuracy and structural fidelity, with final exports in “STEP” file format (Figure 1D).

### 2.5. Building the Model of Post–Core Restoration

The “Measure” tool in SOLIDWORKS (version 29.0.0.5028) was first used to determine the root lengths in German Shepherds, Beagles, and Teddy dogs. A new part file was created, and the sketching environment was accessed via the “Sketch” function. Initial rectangular sketches were converted into fiber post models through the “Extrude” and “Revolve” functions, building post-to-root length ratios of 1/3, 1/2, and 2/3, with 1.8 mm apical diameter, 1.1 mm coronal diameter, and 6% taper [6]. Based on CT measurement data, the alveolar bone model was simplified as a 10 mm × 10 mm × 15 mm rectangular block.

After creating a new assembly in SOLIDWORKS, the alveolar bone, the tooth components, and the post were assembled together, generating one healthy tooth and nine post-restored variants (three post designs × three dog sizes). Finally, each model was opened individually and reconstructed using Boolean operations (“Combine” and “Subtract” functions) within the “Direct Editing” module. The finalized models were exported in Parasolid x_t format (Figure 1E).

### 2.6. Finite Element Simulation of Dental Fracture

Within the ANSYS Workbench (version 17.0.0.19190) interface, the “Static Structural” module was selected to import x_t files of differently sized maxillary canine tooth models. Material properties were assigned in the “Engineering Data” library by editing the required parameters for each material and the specific values are shown in the table below (Table 1), with parameters referenced from the literature [13,14].

Previous research illustrated that the maximum bite force range for canine teeth is 480–1000 N (vertical loading), with tooth fracture thresholds ranging from 294 to 687 N (horizontal loading) [15]. Therefore, this experiment applied progressive loads from 100 N to 1100 N with 100 N increments on both the lingual and distal surfaces. The obtained three-dimensional finite element models were imported into ANSYS software for solution, with total deformation inserted. The total deformation values of the three canine tooth groups under distal and lingual loading from 100 N to 1100 N were compared, and the results were plotted as deformation–load curves (with deformation values on the *x*-axis and load values on the *y*-axis) (Figure 1F).

### 2.7. In Vitro Fracture Resistance Verification

One hundred maxillary canines were extracted from adult dog frozen species, donated to Sichuan Agricultural University Teaching Veterinary Hospital. Among them, 72 teeth without significant wear or defects were selected for the next step. To ensure the reliability and repeatability of the test data, strict quality control procedures were implemented during specimen preparation and mechanical testing. All extracted teeth were inspected under a stereomicroscope to exclude specimens with cracks, root resorption, or structural defects. Only intact, similarly sized teeth were selected and assigned to each group [16]. During specimen embedding, a standardized positioning device was used to ensure uniform angulation and vertical height of each tooth, thereby reducing bias caused by inconsistent installation.

The extracted maxillary canine teeth were immersed in hydrogen peroxide for 12 h to remove residual soft tissues. After drying, the portions were wrapped with elastomeric impression material to create complete tooth molds. The teeth were then sectioned at the cervical level using a dental drill to simulate clinical fractures. The standard root canal procedure was performed. The fiber posts were etched and treated with resin adhesive. After 30 min of polymerization, specimens were mounted in custom fixtures on a universal testing machine. Loading was applied at 1 mm/min on lingual or distal surfaces until fracture occurred [17]. The maximum fracture load (N) and deformation were recorded. Mean values were calculated for each group to generate deformation–load curves (*x*-axis: deformation values, *y*-axis: load values) (Figure 1G). For measurement repeatability, each specimen was tested only once to avoid pre-fatigue damage. The maximum fracture load and deformation value of each sample were recorded automatically by the universal testing machine. All mechanical tests were conducted by the same experienced operator under consistent environmental conditions (temperature 20 ± 3 °C).

The teeth were measured and divided into three size-based groups (*n* = 24 each): B group (4.7–5.1 cm), M group (3.9–4.2 cm), and S group (2.9–3.3 cm). Each group contained control subgroups (Bc, Mc, Sc) and experimental subgroups with varying post-to-root length ratios (B1/M1/S1:1/3; B2/M2/S2:1/2; B3/M3/S3:2/3; *n* = 6 per subgroup).

### 2.8. Statistical Analysis

Using the fracture resistance results from healthy canine teeth (BC, MC, SC) as reference values for distal–caudal and lingual–buccal loading, maximum principal stress (σ_1_, the basis for analyzing the fracture risk) and minimum principal stress (σ_2_, for analyzing the collapse risk due to compression) as failure indices were analyzed according to the Maximum Principal Stress Criterion (MPSC) using the ANSYS “Solution” function. Pearson correlation coefficients were calculated to assess the correlation of the total deformation values between finite element analysis (FEA) and in vitro fracture. All statistical analyses were performed using SPSS 26.0 (IBM Corp., Armonk, NY, USA). Data were expressed as mean ± standard deviation (SD). A significance level of *p* < 0.05 was considered statistically significant.

## 3. Results

### 3.1. Total Deformation Analysis of Upper Canine Teeth in Different Canine Sizes Using FEA

**Figure 2:**
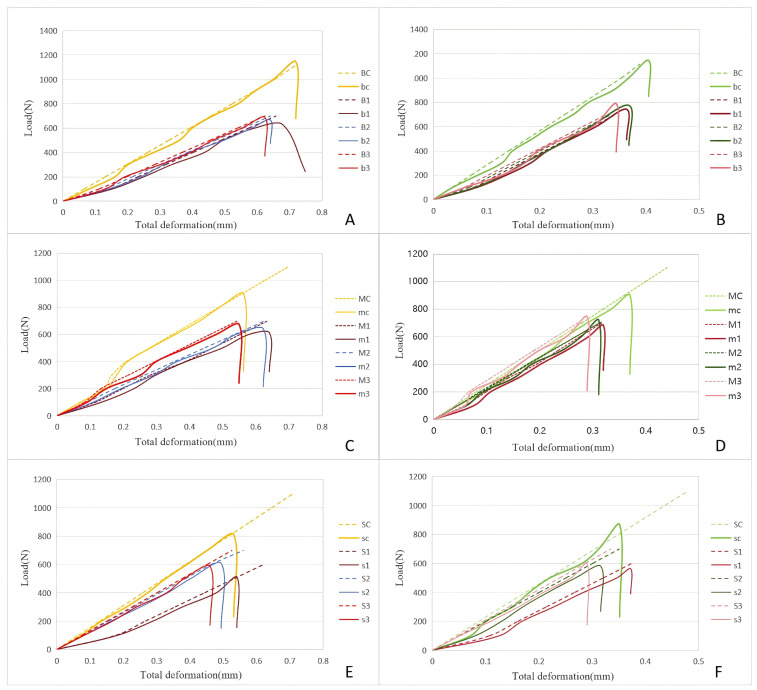
Total deformation of big (**A**,**B**), medium (**C**,**D**), and small (**E**,**F**) dogs from distal–caudal (**A**,**C**,**E**) and lingual–buccal (**B**,**D**,**F**) directions under loads. Dotted line: FEA result; Solid line: universal testing machine results.

The total deformation under distal loading exceeded that of lingual loading across all groups. For healthy canines, large-breed dogs (BC) showed maximum deformations of 0.71915 mm (distal–caudal) and 0.38879 mm (lingual–buccal), medium breeds (MC) exhibited 0.56275 mm (distal–caudal) and 0.368325 mm (lingual–buccal), while small breeds (SC) displayed 0.51568 mm (distal–caudal) and 0.34951 mm (lingual–buccal). In the 2/3 root-to-post ratio restoration groups, large breeds (B3) demonstrated maximum deformations of 0.6248 mm (distal–caudal) and 0.32538 mm (lingual–buccal), medium breeds (M3) showed 0.54495 mm (distal–caudal) and 0.272 mm (lingual–buccal), and small breeds (S3) presented 0.45137 mm (distal–caudal) and 0.28714 mm (lingual–buccal). Trend analysis of the curves (Figure 2 dotted lines) revealed a consistent linear progression in total deformation values calculated through finite element analysis.

### 3.2. Total Deformation of Upper Canine Teeth in Different Canine Sizes Measured by Universal Testing Machine

The results obtained from the universal testing machine showed strong agreement with finite element analysis (FEA) findings, with distal loading consistently producing greater total deformation than lingual loading across all test groups. In healthy canines, large-breed dogs (BC) exhibited maximum deformation values of 0.7191 mm (distal–caudal) and 0.38879 mm (lingual–buccal), medium breeds (MC) demonstrated values of 0.55849 mm (distal–caudal) and 0.3681 mm (lingual–buccal), while small breeds (SC) showed values of 0.531075 mm (distal–caudal) and 0.35165 mm (lingual–buccal). Among the restoration groups with a 2/3 root-to-post ratio, large breeds (B3) displayed maximum deformations of 0.62355 mm (distal–caudal) and 0.343875 mm (lingual–buccal), medium breeds (M3) recorded 0.549165 mm (distal–caudal) and 0.2894 mm (lingual–buccal), and small breeds (S3) presented 0.460025 mm (distal–caudal) and 0.29055 mm (lingual–buccal). Trend analysis of the curves (Figure 2, solid lines) revealed that total deformation plateaued while fracture resistance dropped abruptly at the point of tooth fracture.

### 3.3. Fracture Resistance of Upper Canine Teeth Across Canine Size Variants in Universal Testing Machine Measurements

The universal testing machine results (Table 2) demonstrate the fracture resistance under lingual loading compared to distal loading across all canine sizes, with large-breed dogs exhibiting the highest values. Post–core restorations significantly reduced fracture resistance in all size groups, though the 2/3 root-to-post ratio restoration groups (B3, M3, S3) showed fracture resistance levels closest to those of healthy controls (BC, MC, SC), with the smallest reduction rates. The fracture resistance values for BC, MC, and SC were 1115.851 N (distal–caudal) and 1177.39 N (lingual–buccal), 901.627 N (distal–caudal) and 976.504 N (lingual–buccal), and 812.733 N (distal–caudal) and 897.642 N (lingual–buccal), respectively. The B3, M3, and S3 groups reduction rates were 38.23%, 25.19%, and 27.58% under distal–caudal loading, and 33.86%, 23.73%, and 33.24% under lingual–buccal loading, respectively (Table 2).

The 2/3 post-to-root ratio groups (B3, M3, S3) showed significantly higher fracture loads than the 1/3 and 1/2 groups in all body sizes (*p* < 0.05). Large-breed dogs exhibited significantly higher fracture resistance than medium and small breeds under both distal and lingual loading (*p* < 0.05).

### 3.4. Correlation Coefficients of Total Deformation

The correlation coefficients between total deformation measurements from universal testing machine results and FEA predictions illustrated that all three experimental groups demonstrated correlation coefficients ranging from 0.9257 to 0.9779, demonstrating a strong correlation (Table 3). These results consistently indicate a highly significant correlation between FEA predictions and experimental measurements, confirming that finite element analysis provides sufficiently reliable predictions for in vitro biomechanical testing of canine tooth models.

The correlation coefficients between FEA and in vitro tests ranged from 0.9257 to 0.9779, indicating a strong positive correlation (*p* < 0.01).

### 3.5. Maximum (σ1) and Minimum (σ2) Principal Stresses in Upper Canines Across Canine Sizes

**Figure 3:**
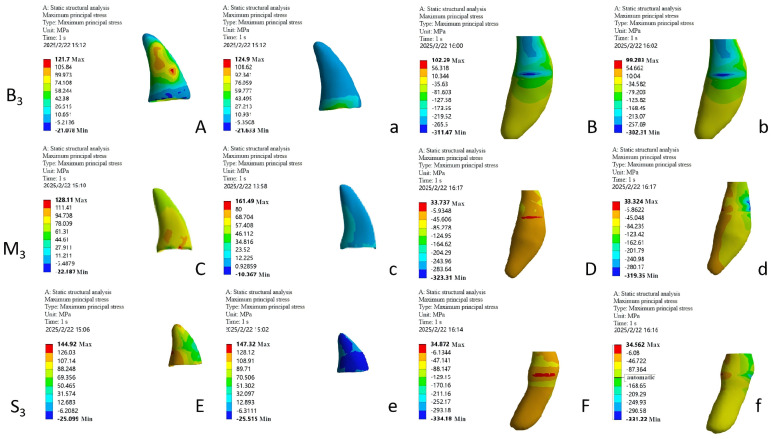
σ_1_ and σ_2_ contours with different loads at a root-to-post ratio of 2/3 under distal–caudal (**A**–**F**) and lingual–buccal (**a**–**f**) directions. B3: Large dogs; M3: medium-sized dog; S3: Small dogs.

Using the actual fracture forces measured by the universal testing machine, the σ_1_ (crown) and σ_2_ (root) stress were calculated by FEA (Table 4). Taking 2/3 root–post ratio test groups for example (Figure 3), under distal–caudal loading, crown stresses were primarily concentrated at the mesial and distal cervical regions, while root stresses were mainly focused at the dentin enamel junction, with more extensive red zones observed in B1, M1, and S1 models. Under lingual–buccal loading, crown stresses were predominantly localized at the lingual cervical region, while root stresses were distributed widely along the entire lingual root surface.

Additionally, there is a consistent decreasing trend in both σ_1_ and σ_2_ stress values across all three experimental groups under distal–caudal loading (Table 4). The failure indices for crowns and roots in all test groups exceeded group 1, indicating fracture occurrence in these components. The B3, M3, and S3 groups demonstrated the lowest resin σ_1_ values (121.7 MPa, 128.11 MPa, and 144.92 MPa respectively), with failure indices of 1.581, 1.664, and 1.882. Similarly, these groups showed minimal σ_2_ peak values (−311 MPa, −323.31 MPa, and −334.18 MPa), with failure indices of 1.047, 1.089, and 1.125, respectively.

Similarly to distal–caudal loading, on lingual–buccal loading both σ_1_ and σ_2_ stresses demonstrated a decreasing trend across all three experimental groups under lingual loading conditions. The groups B3, M3, and S3 showed the lowest resin σ_1_ values of 124.9 MPa, 131.31 MPa, and 147.32 MPa, corresponding to failure indices of 1.622, 1.705, and 1.913, respectively. Similarly, these groups displayed minimal σ_2_ peak stresses of −302.31 MPa, −319.35 MPa, and −331.22 MPa with failure indices measuring 1.018, 1.075, and 1.115, respectively (Table 5). These results demonstrate that regardless of canine size, the 2/3 root-to-post ratio configuration consistently provides enhanced fracture resistance for both crowns and root components.

## 4. Discussion

Post–core restoration technology remains essential for restoring extensively damaged teeth. Controversy persists regarding fiber post length effects. For example, Marinescu et al. [18] found there is no statistical correlation with post-to-root length ratios, while Lin et al. [5] and Amarnath et al. [6] reported increased fracture resistance with longer posts. From an engineering perspective, increased post length reduces stress by increasing bending moment [19]. Clinically, slightly shorter posts may be necessary when ideal lengths compromise the apical seal [18], emphasizing conservative preparation to preserve tooth structure [20]. In the present study, we sought to identify the optimal post–core proportion for canine teeth in dogs by employing both finite element analysis and universal testing machine measurements.

For the feasibility of computational analysis, in the finite element analysis scenario of this study, the following assumptions were made to ensure consistency and computational feasibility: (a) Perfect bonding is assumed for all components within the modeled assembly, meaning no interfacial gaps or micro-movements exist between adjacent structures. (b) The cross-sectional surfaces of cancellous and cortical bone are fully constrained to prevent any translational or rotational displacement, thereby simulating fixed boundary conditions. (c) The normal pre-stress that may be generated during root canal treatment is ignored, assuming its contribution to the overall stress distribution is negligible. (d) The geometric model of the maxillary central incisor restored with a post-and-core system and a porcelain-fused-to-metal crown is idealized to simplify the analysis without compromising clinical relevance. (e) All materials used in the tooth model are defined as homogeneous, isotropic, and linearly elastic, in accordance with widely accepted conventions in dental biomechanical modeling [21].

Canine tooth morphology correlates strongly with body weight and size. The characteristic lingual curvature of canines enhances prey retention during hunting. Under loading, more circular cross-sections demonstrate superior stress distribution, while higher ellipticity improves tissue penetration [22]. Our in vitro force results confirmed large breeds’ superior fracture resistance (distal–caudal: 1144.851 N, lingual–buccal: 1177.39 N) compared to medium (distal–caudal: 901.627 N, lingual–buccal: 976.504 N) and small breeds (distal–caudal: 812.733 N, lingual–buccal: 897.642 N). Of note, while FEA effectively simulates occlusal mechanics, real-world complexities like periodontal ligament (PDL) anisotropy were simplified to isotropic linear elasticity in our model. Given PDL’s nonstandard thickness and the clinical reality that mobility only occurs with ≥6 mm periodontal damage, we omit PDL, similarly to previous research [19]. Universal testing machine validation confirmed our FEA predictions. Both methods yielded comparable deformation patterns under progressive loading. Our results consistently indicate a highly significant correlation between FEA predictions and experimental measurements, confirming that FEA provides sufficiently reliable predictions for in vitro biomechanical testing of canine tooth models. This mechanical testing remains indispensable for determining absolute fracture strengths and validating computational models in dental research.

Finite element analysis (FEA) results showed both σ_1_ and σ_2_ concentrations at the cervical region, indicating highest fracture risk—consistent with most similar studies [23]. This phenomenon may stem from structural weaknesses at the cementoenamel junction where stress transmission is inefficient. Additionally, the canine tooth’s high crown-to-root ratio and elongated root allow longer fiber posts to better distribute stress apically. Our data demonstrated failure indices for B3/M3/S3 groups under distal loading at 1.581/1.664/1.882 (crown) and 1.047/1.089/1.125 (root), and under lingual loading at 1.622/1.705/1.913 (crown) and 1.018/1.075/1.115 (root), with σ_1_ concentrating at mesial/distal cervical areas—aligning with findings from the literature. Therefore, clinicians should preserve mesial/distal tooth structure during preparation to minimize fracture risk.

Although the 2/3 post-to-root ratio group showed statistically superior fracture resistance and lower stress values compared with the 1/3 and 1/2 groups, it is essential to distinguish statistical significance from clinical relevance. The observed differences in fracture load and stress distribution reflect biomechanical advantages under experimental conditions, yet their clinical impact depends on multiple practical factors, including surgical operation difficulty, residual tooth structure, root canal anatomy, and long-term maintenance [24,25]. Therefore, the clinical application of these findings should be prudent and individualized rather than purely mechanically guided. The goal of restorative dentistry is not only to maximize fracture strength but also to preserve remaining tooth tissue, maintain periodontal health, and ensure long-term functional stability [25]. The present study offers quantitative biomechanical evidence and a clinically applicable ratio (2/3) that can directly guide post selection. This work fills the knowledge gap regarding standardized restoration protocols for dog canine teeth and supports the development of evidence-based veterinary restorative dentistry. By linking laboratory data to clinical decision-making, this study enhances the translational value of basic biomechanical research and promotes more predictable and reliable restorative outcomes.

Several limitations must be acknowledged to improve the scientific rigor and generalizability of the conclusions. First, all materials were modeled as isotropic, homogeneous, and linearly elastic, which simplifies the anisotropic and viscoelastic behavior of biological tissues (dentin, enamel, bone). Second, the periodontal ligament (PDL) was omitted, and the alveolar bone was simplified as a regular cube. While this omission is common in similar studies [19] and justified by the minimal tooth mobility in healthy dogs, it may affect stress damping near the cervical region. Third, only static loading was applied; the oral environment involves cyclic fatigue, thermal changes, and variable occlusal forces. Fourth, the in vitro fracture test used extracted teeth without a simulated periodontal environment, which may underestimate the energy-absorbing capacity of the tooth–bone complex. Fifth, the sample size per group (*n* = 6) was relatively small, although statistical significance was still achieved. Finally, this study used only one tooth type (maxillary canine) from three specific breeds; results may not generalize to other teeth or mixed-breed dogs with different root morphologies.

## 5. Conclusions

In conclusion, a post-to-root length ratio of 2/3 provides the best biomechanical outcome for fiber post–core crown restoration of fractured maxillary canine teeth in dogs, regardless of body size. Clinicians might prioritize preserving cervical tooth structure and select a post length of approximately 2/3 of the root length whenever anatomically feasible. However, individual variations in root curvature, canal diameter, and remaining coronal dentin must be considered. Future in vivo long-term studies are needed to confirm these findings.

## Figures and Tables

**Table 1:** Mechanical properties of each component.

Material	Elastic Modulus (MPa)	Poisson’s Ratio
Cortical bone	13,700	0.30
Trabecular bone	1370	0.30
Dentin	18,600	0.31
Enamel	84,000	0.33
Dental pulp	2	0.33
Gutta-percha	141.9	0.45
Resin crown	18,300	0.30
Fiber post	15,000	0.30

**Table 2:** Average bending resistance of canine teeth.

Model	Distal Loading (N)	Lingual Loading (N)
B_C_	1115.851 ± 6.984	1177.39 ± 5.82
B_1_	631.229 ± 4.45	743.827 ± 8.179
B_2_	667.023 ± 6.49	775.146 ± 6.2
B_3_	689.264 ± 8.447	778.691 ± 8.415
M_C_	901.627 ± 7.49	976.504 ± 6.399
M_1_	603.175 ± 5.4	682.249 ± 6.411
M_2_	642.824 ± 6.501	719.276 ± 4.4
M_3_	674.467 ± 5.48	744.823 ± 5.402
S_C_	812.733 ± 5.476	897.642 ± 6.42
S_1_	511.073 ± 5.492	563.864 ± 5.4
S_2_	551.964 ± 4.498	609.287 ± 4.507
S_3_	588.618 ± 5.489	599.257 ± 4.507

**Table 3:** Correlation coefficient between FEA and total deformation of the universal testing machine.

Model	Distal Loading Correlation Coefficient	Lingual Loading Correlation Coefficient
B_C_	0.9779	0.9486
B_1_	0.9775	0.9586
B_2_	0.9654	0.9604
B_3_	0.9634	0.9768
M_C_	0.9733	0.9534
M_1_	0.9594	0.9651
M_2_	0.9792	0.964
M_3_	0.9602	0.9257
S_C_	0.9604	0.9583
S_1_	0.9613	0.9507
S_2_	0.9648	0.9681
S_3_	0.9585	0.9497

**Table 4:** σ_1_, σ_2_ and failure indices of experimental groups under distal loading.

Model Group	Crown σ_1_ (MPa)	Root σ_2_ (MPa)	Failure Index 1	Failure Index 2
B_1_	164.96	−338.14	2.142	1.139
B_2_	158.01	−328.25	2.052	1.105
B_3_	121.7	−311	1.581	1.047
M_1_	174.55	−348.12	2.267	1.172
M_2_	166.69	−333.19	2.165	1.122
M_3_	128.11	−323.31	1.664	1.089
S_1_	191	−355.93	2.481	1.1988
S_2_	173.64	−344.07	2.255	1.158
S_3_	144.92	−334.18	1.882	1.125

Failure Index = σ_1_/σTS or σ_2_/σCS; Failure Index 1 represents the crown failure index; Failure Index 2 represents the root failure index.

**Table 5:** σ_1_, σ_2_ and failure indices of experimental groups under lingual loading.

Model Group	Crown σ_1_ (MPa)	Root σ_2_ (MPa)	Failure Index 1	Failure Index 2
B1	168.43	−334.18	2.187	1.125
B2	163.22	−321.33	2.12	1.082
B3	124.9	−302.31	1.622	1.018
M1	176.15	−348.03	2.288	1.172
M2	161.49	−329	2.097	1.108
M3	131.31	−319.35	1.705	1.075
S1	186.66	−353.96	2.424	1.192
S2	171.34	−340.12	2.225	1.145
S3	147.32	−331.22	1.913	1.115

Failure Index = σ_1_/σTS or σ_2_/σCS; Failure Index 1 represents the crown failure index; Failure Index 2 represents the root failure index.

## Data Availability

The original contributions presented in this study are included in the article. Further inquiries can be directed to the corresponding authors.

## Abbreviation

FEA	Finite Element Analysis
CT	Computed Tomography
DICOM	Digital Imaging and Communications in Medicine
STL	Stereolithography
STEP	Standard for the Exchange of Product model data
σ_1_	Maximum Principal Stress
σ_2_	Minimum Principal Stress
MPSC	Maximum Principal Stress Criterion
PDL	Periodontal Ligament
B	Large-breed dogs
M	Medium-breed dogs
S	Small-breed dogs
BC	Large-breed control group
MC	Medium-breed control group
SC	Small-breed control group
B1/M1/S1	Post-to-root ratio 1/3 group
B2/M2/S2	Post-to-root ratio 1/2 group
B3/M3/S3	Post-to-root ratio 2/3 group
N	Newton
MPa	Megapascal
mm	Millimeter
